# Sinonasal Phosphaturic Mesenchymal Tumor Presenting With Tumor-Induced Osteomalacia: A Rare Case and Review of the Literature

**DOI:** 10.7759/cureus.113825

**Published:** 2026-08-02

**Authors:** Shouvanik Satpathy, Devmalya Banerjee, Subhadeep Das, Dulal Bose, Kamalendu Halder

**Affiliations:** 1 Department of Otolaryngology - Head and Neck Surgery, Peerless Hospital and B.K. Roy Research Center, Kolkata, IND; 2 Department of Histopathology and Laboratory Services, Peerless Hospital and B.K. Roy Research Center, Kolkata, IND

**Keywords:** mesenchymal, osteomalacia, paraneoplastic syndrome, phosphaturia, sinunasal mass

## Abstract

Phosphaturic mesenchymal tumor (PMT) is a rare tumor involving bone and soft tissues, usually benign and solitary. They mainly affect the extremities and are rarely seen in the head and neck region. They are most commonly associated with tumor-induced osteomalacia (TIO). TIO, also known as oncogenic osteomalacia, is a paraneoplastic syndrome in which tumor cells secrete hormones that reduce mineralization of mature bone, thereby decreasing osteoblastic activity and causing renal phosphate wasting. One of the most common hormones involved is fibroblast growth factor-23 (FGF-23), which belongs to a group of *phosphatonins*. These tumors are seen in middle-aged individuals and present with symptoms and signs suggestive of stress fractures. The investigative modalities include blood investigations, such as serum FGF23, which is highly suggestive of PMT. Serum phosphorus levels are also indicative. Conventional imaging reveals multiple insufficiency fractures and decreased osseous density. Molecular imaging, such as positron emission tomography/computed tomography (PET/CT), can help detect occult lesions. The confirmatory test is histopathology. Once diagnosed, the treatment of choice is surgical excision. We present a case of a 61-year-old male who presented with multiple stress fractures and was initially treated for arthropathy. However, on further investigations, a diagnosis of sinonasal PMT was made, and the tumor was removed surgically. Post-surgery, the patient improved in terms of symptoms and biochemical parameters, with no paraneoplastic syndrome. The histopathology report confirmed the diagnosis.

## Introduction

Phosphaturic mesenchymal tumor (PMT) is a rare tumor involving bone and soft tissues, usually benign and solitary in nature. They are found mostly in the extremities and appendages, with head and neck involvement seen in hardly 5% of the cases [1,2]. More than half of head-and-neck PMTs are seen in the sinonasal areas [3]. It is a tumor that is most commonly associated with tumor-induced osteomalacia (TIO) [4].

TIO, or oncogenic osteomalacia, is a paraneoplastic syndrome in which tumor cells secrete certain hormones, e.g., fibroblast growth factor-23 (FGF-23) in PMTs, that cause reduced mineralization of mature bone and renal phosphate wasting. One of the most common hormones involved is FGF-23, a member of the “phosphatonins” family [5]. FGF-23 is a hormone that reduces phosphate reabsorption from the proximal tubules and inhibits an enzyme, 1-α-hydroxylase, that ultimately reduces levels of 1-α, 25-dihydroxy vitamin D3. Therefore, in patients with PMT, there is overexpression of FGF-23, leading to increased urinary phosphate clearance, calcium and phosphate mobilization from bone, and reduced osteoblastic activity. These pathological changes lead to osteomalacia [6,7]. This tumor occurs in middle-aged individuals and presents with symptoms and signs such as fractures, gait disturbances, weakness, or bone pain, all suggestive of demineralization of bone [7].

As seen in routine practice and the literature, there are very few cases of PMTs seen in the head and neck area. It was first described in 1987 by Weidner and Santa Cruz, who named it for its polymorphous histology [8]. Since then, there have been reports of this benign tumor, though not commonly, from the head and neck area.

Hence, we present an interesting case of PMT in the sinonasal region with TIO that is very rare, proved extremely challenging to diagnose, and required several months to reach a diagnosis.

## Case presentation

Our patient, a 61-year-old Indian male from the northeastern part of India, presented with repeated stress fractures, generalized body pain, weakness, and difficulty walking since January 2024. There were multiple stress fractures involving various areas of the bony skeleton, ranging from the femur to the metatarsals, over one year.

He was diagnosed with hypertension in 2024, with no other comorbidities. He had undergone extensive evaluation by multiple clinicians due to chronic symptoms and was finally treated for seronegative arthropathy up to June 2025 with methotrexate, sulfasalazine, etc. Erythrocyte sedimentation rate (ESR), C-reactive protein (CRP), anti-cyclic citrullinated peptide (anti-CCP), rheumatoid factor (RF), and human leukocyte antigen B27 (HLA-B27) were all normal, and antinuclear factor (ANF) was borderline high.

He was treated with teriparatide (synthetic human parathyroid hormone) from November 2023 to January 2024 and from December 2024 to February 2025. He was also started on calcium supplements. Due to ongoing recurrent fractures, he was evaluated further.

A series of investigations was performed, including blood tests and nuclear imaging, to establish the diagnosis. Serum phosphate (0.5 mg/dL) and serum calcium (7.8-9 mg/dL) were low, while alkaline phosphatase (132 U/L), plasma parathormone (395.5 pg/mL), and FGF-23 (472.4 pg/mL) were markedly elevated. Chemiluminescence immunoassay was used to measure the FGF-23 level (Table 1). Therefore, PMT was suspected.

**Table 1:** Different blood parameters suggestive of phosphaturic mesenchymal tumor.

Blood parameters	Obtained values	Normal range
Serum calcium	7.8 mg/100 mL	8.7-10.2 mg/100 mL
Serum phosphorus	0.5 mg/100 mL	2.5-4.5 mg/100 mL
Alkaline phosphatase	132 U/L	Adult male: 40-130 U/L; adult female: 35-105 U/L
Plasma parathormone	395.5 pg/mL	15-65 pg/mL
Fibroblast growth factor-23 (FGF-23)	472.4 pg/mL	23.2-95.4 pg/mL
Serum vitamin D	37.8 ng/mL	>30 ng/mL

**Figure 1:**
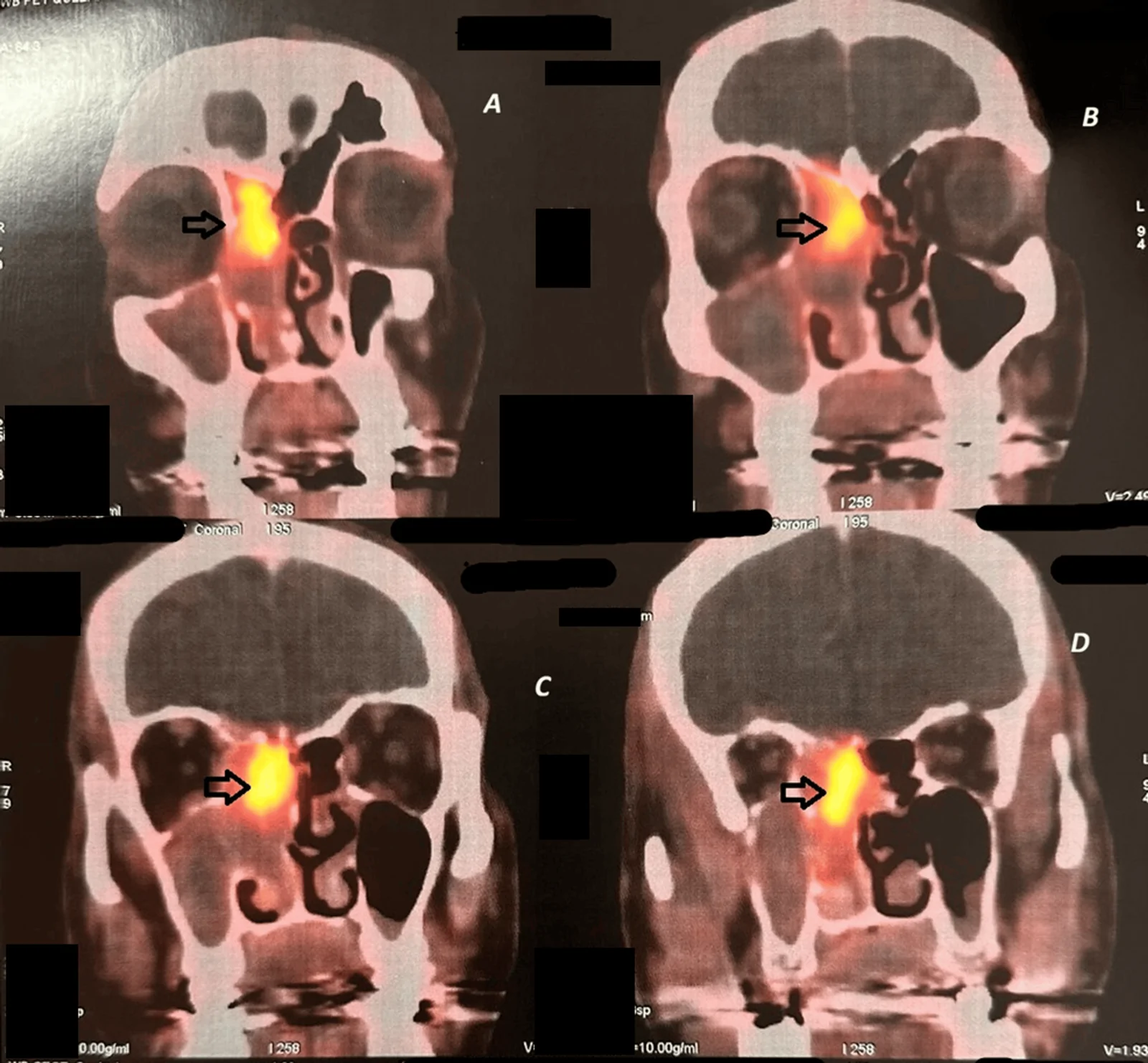
Gallium-68 scan in coronal cuts of the paranasal sinuses showing an enhancing mass extending superiorly into the right frontal sinus (arrow) (A, B), involving the right ethmoid sinus (arrow) (C), and extending below into the nasal cavity (arrow) (D).

MRI of the brain indicated the potential presence of an infiltrative intranasal mesenchymal neoplasm. An FDG whole-body PET scan was performed. Tracers such as gallium-68 (68Ga) tetraazacyclododecane-tetraacetic acid-octreotate (DOTATATE), a somatostatin analog, were used, as PMTs are known to express somatostatin receptors. On the FDG-PET scan, a somatostatin receptor hyper-expressing enhancing soft tissue mass measuring approximately 1.8 × 4.2 × 2.6 cm, with an SUV max of 9.4, was found in the right ethmoid air cell, contiguously extending to the frontal sinus superiorly and into the nasal cavity inferiorly (Figure 1).

**Figure 2:**
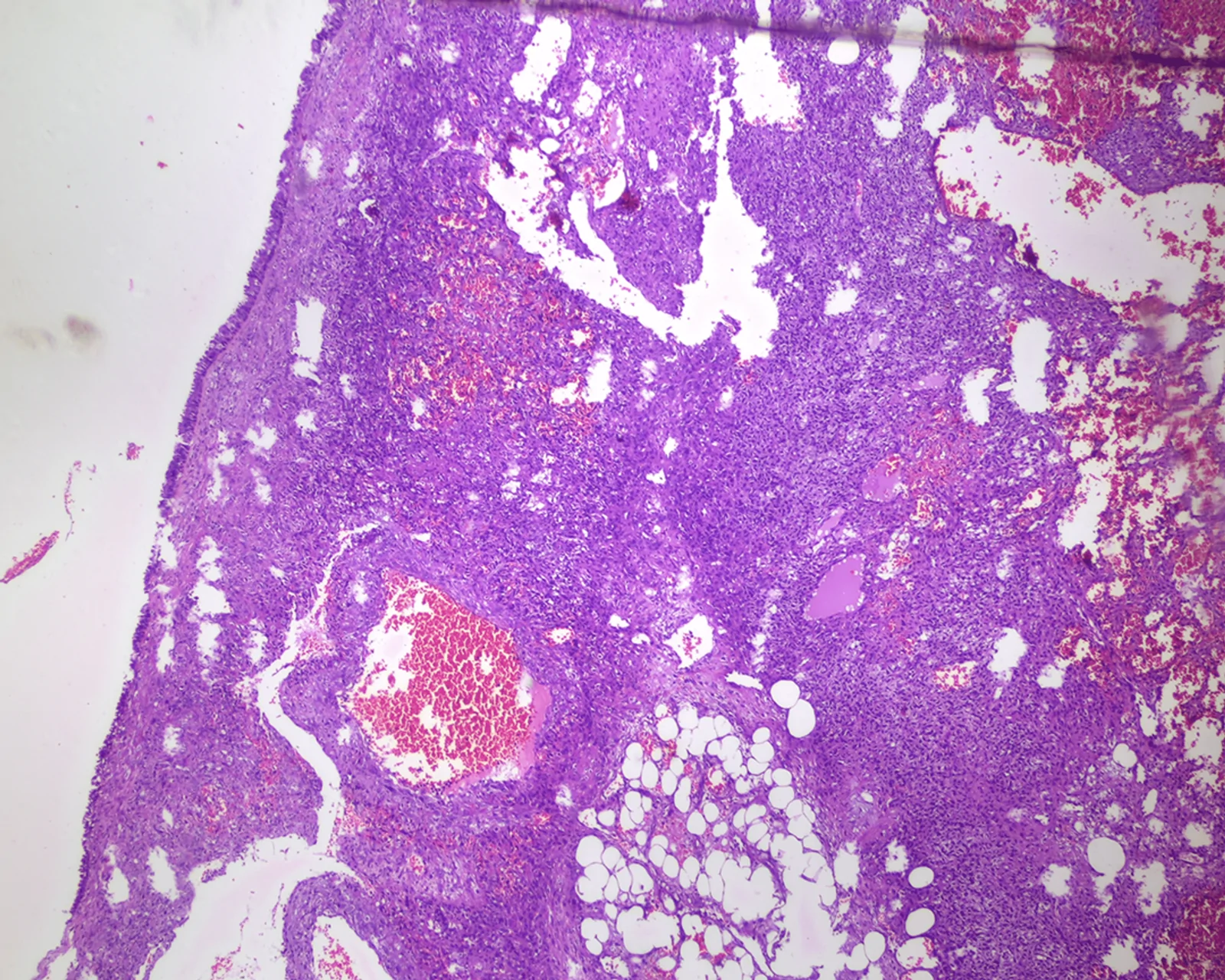
Histopathological examination showing a hemangiopericytoma-like vascular pattern with characteristic grungy calcification.

Endoscopic biopsy was taken from the lesion and revealed a PMT of the paranasal sinuses. It showed a prominent hemangiopericytoma-like vascular pattern with characteristic grungy calcification (Figure 2).

**Figure 3:**
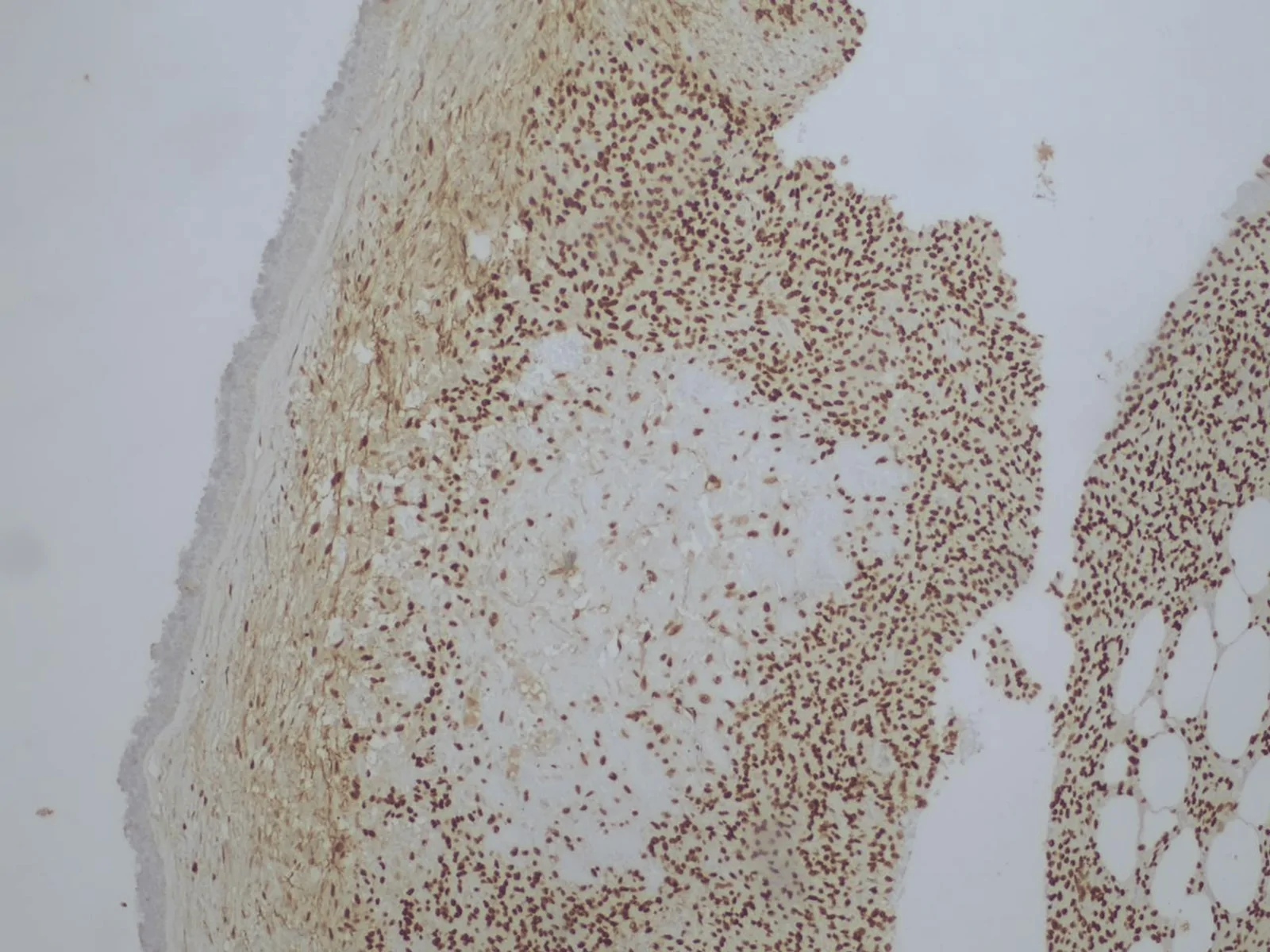
Immunohistochemistry showing tumor cells with cytoplasmic and membranous positivity for CD56 and nuclear positivity for SATB2.

On immunohistochemistry, tumor cells showed cytoplasmic and membranous positivity for cluster of differentiation 56 (CD56) and nuclear positivity for SATB2 (Figure 3).

ERG was focally but distinctly positive. The tumor cells were negative for EMA, S100, SOX10, Cyclin D1, cytokeratin, and STAT6. Therefore, a diagnosis of PMT was confirmed.

Upon confirmation of the diagnosis, the PMT was surgically excised. An endoscopic approach with the Coblator was employed. Angled telescopes aided in peripheral dissection and maintenance of hemostasis. The Coblator was used for both dissection and control of bleeding. The mass involved the right ethmoid sinus and right nasal cavity, extending superiorly into the frontal sinus. It was observed to infiltrate the right maxillary sinus. The medial extent reached the left ethmoid sinus. Part of the mass was pressing against the medial orbital wall of the right eye. The mass was removed piecemeal from the right ethmoid and frontal sinuses, as well as the orbital wall, using the Coblator in critical areas. The entire mass was removed without residual tissue, as confirmed by endoscopy. Frozen section was not performed as the mass was removed piecemeal. The operative time was around three hours without any intraoperative complications, such as breach of the skull base or cerebrospinal fluid (CSF) leak.

The postoperative period was uneventful. Post-surgery, the nasal pack was removed on postoperative day 2, and the patient was discharged. His phosphate level returned to normal in the immediate postoperative period.

The final histopathology report also confirmed the diagnosis of PMT.

The patient was on regular follow-up, with no recurrence or residual tumor at one and three months, as confirmed by diagnostic nasal endoscopy. He improved clinically, with relief of bone pain and weakness, was able to walk normally, and previous fractures had healed with no further fractures.

## Discussion

These tumors arise from any area containing bone and soft tissue but are extremely rare in the head and neck region. A review study by Deep et al. found 33 case reports of head and neck cases between 1970 and 2013. In more than half the cases (17), the PMTs were found in the head and neck region, whereas in 16 cases, they were found in the pharynx, larynx, thyroid, mandible, floor of the mouth, and temporal bone [3]. As per various studies in the literature, there are about 153 cases of PMT found in the sinonasal region, and they are most commonly located in the ethmoid sinus (64.7%), as seen in our case, followed by the nasal cavity, maxillary sinus, frontal sinus, and sphenoid sinus in 50.3%, 19%, 16.4%, and 11.8%, respectively [9].

These tumors can be easily misdiagnosed due to their varied presentations, especially when they involve the head and neck region. The mean age at diagnosis is around 44-48 years [10,11]. Most of these may even go unrecognized for years and be treated as arthropathy or other causes of osteoporosis for some time, as seen initially in our case. As seen in our case, PMTs are seen in middle-aged men and present with recurrent stress fractures.

Therefore, the use of basic investigations that can help aid in diagnosis should be considered in a patient with multiple stress fractures. Serum phosphate levels and creatinine levels can give a hint; however, a rise in serum FGF-23 levels is diagnostic. Conventional imaging modalities reveal multiple insufficiency fractures and decreased osseous density. On CT, osteolytic defects with a narrow transition zone and some internal matrix may be seen, although PMTs are hypodense and take up contrast. On MRI, there is post-contrast enhancement, and the lesion is isointense to soft tissues on T1-weighted images. The T2-weighted images appear hyperintense, though variations can occur. Molecular imaging, such as PET-CT, is also helpful in detecting lesions, including occult ones. Somatostatin-based radiotracers, such as 68Ga-DOTATATE, are applicable as PMTs express somatostatin receptors [12]. However, the sensitivity of all these tests varies across a series by El-Maouche et al. [13].

A biopsy with histopathological examination helps clinch the diagnosis. Satellite cells or spindle cells with low nuclear grade are seen in a myxochondroid or myxoid matrix with flocculent or grungy calcification. A rich microvascular supply, similar to that seen in hemangiopericytoma, is characteristic of PMTs [3]. On immunohistochemistry, tumor cells show cytoplasmic and membranous positivity for CD56 and nuclear positivity for SATB2. ERG is focally but distinctly positive. The tumor cells are negative for EMA, S100, SOX10, Cyclin D1, cytokeratin, and STAT6.

The diagnostic challenges increase due to the diversity of tumor locations. Therefore, biopsy with specific nuclear imaging modalities, as well as a strong suspicion of tumor-induced osteomalacia, gives a hint toward diagnosing such tumors.

The only known treatment so far is surgical excision of the mass. A complete cure is seen in 90% of cases. To avoid any residual tumor or recurrence, wide excision of the mass is usually performed. Other modalities, such as cryoablation, have also been successfully used to treat recurrence. Coblation was used in our case to perform meticulous bloodless dissection of the tumor from its peripheries, and no recurrence was observed during follow-up. In inoperable cases, oral phosphate can be tried, and recently, trials of image-guided tumor ablation and the anti-FGF-23 monoclonal antibody KRN23 are being used [12]. A human monoclonal antibody against FGF-23, Burosumab, has recently been approved by the FDA for the treatment of PMT [14]. The paraneoplastic syndrome resolves with tumor resection, and all biochemical parameters return to normal. Full recovery is fortunately seen in most treated cases of PMT.

## Conclusions

PMTs are sporadic tumors of the head and neck region that can present with bony changes and paraneoplastic syndrome. They are associated with tumor-induced osteomalacia, thereby contributing to weakness and bony pain. Although the location in the head and neck region is quite rare, PMTs can be diagnosed with high clinical suspicion, supported by investigations such as FGF-23 levels, serum phosphorus, PET/CT, other molecular imaging modalities, and MRI. Biopsy and histopathological examination confirm the diagnosis. The treatment is surgical excision, which normalizes biochemical parameters and has a good prognosis. Therefore, PMTs are rare and unusual tumors that should not be mistaken for other causes of osteoporosis and require immediate attention.
